# Omalizumab in children with severe allergic disease: a case series

**DOI:** 10.1186/s13052-019-0602-5

**Published:** 2019-01-14

**Authors:** Giuseppe Crisafulli, Lucia Caminiti, Fernanda Chiera, Stefania Arasi, Giuseppina Salzano, Ilenia Panasiti, Andrea Barbalace, Giovanni Battista Pajno

**Affiliations:** 1 Department of Pediatrics, Allergy Unit, University of Messina, Via Consolare Valeria-Gazzi, 98124 Messina, Italy; 2 Department of Pediatrics, Pediatric Unit, San Giovanni di Dio Hospital, Crotone, Italy; 3 Pediatric Allergology Unit, Bambino Gesù Hospital (IRCCS), Rome, Italy

**Keywords:** Omalizumab. Asthma. Food allergy. Immunoglobulin E. pediatrics

## Abstract

**Background:**

Currently, severe allergic asthma and food allergy in children represent an important public health problem with medical, psychosocial and economic impacts. Omalizumab is a humanized monoclonal anti-IgE antibody, approved for refractory allergic asthma and chronic urticaria. It has been widely used in clinical practice as add-on therapy in patients with severe uncontrolled allergic asthma. In recent years there has seen the emergence of an allergic epidemic with increasing food allergy, which represents the main cause of anaphylaxis in children. The standard of care for food allergy is strictly dietary allergen avoidance and emergency treatment, but recent clinical trials have suggested that omalizumab may have a role to play as an adjuvant to oral immunotherapy (OIT). We present a case series of patients treated at our institution with omalizumab for severe allergic asthma and food allergy.

**Methods:**

Patients received omalizumab according to a standard reference nomogram after failing standard therapies. In children with comorbid severe food allergy, omalizumab was administered in conjunction with an oral immunotherapy protocol.

**Results:**

Omalizumab was effective in controlling symptoms of allergic asthma, allergic rhinitis and rhinosinusitis, but not eosinophilic esophagitis, while aiding successful oral desensitization of comorbid severe food allergies.

**Conclusions:**

Omalizumab appears to be an excellent therapeutic option in children with inadequately controlled severe allergic asthma, allergic rhinitis and rhinosinusitis, with or without food allergy.

## Backgrouds

Omalizumab is a recombinant monoclonal antibody (mAb) that binds to the Fcε portion of the immunoglobulin (Ig)E antibodies, reducing total IgE levels, preventing interaction with the high-affinity receptors (FcεRI) expressed on the surface of target cells, receptor expression and interrupting the resulting inflammatory cascade [1].

Omalizumab has been licensed since 2003 by the Food and Drug Administration (FDA) for the treatment of moderate-to-severe allergic asthma in adults and adolescents (≥12 years) with sensitization to a perennial allergen and symptoms not adequately controlled by inhaled corticosteroids (ICS) [2]. In 2005, the European Medicines Agency (EMA) approved it as add-on therapy for the treatment of persistent severe allergic asthma not controlled by daily high-dose inhaled corticosteroids (ICS) plus inhaled long-acting β2-agonist (LABA) therapy in adults and adolescents (age ≥ 12 years) [3]. Omalizumab was subsequently approved by the EMA [4] and the FDA [2] in children aged 6 to 12 years with severe persistent allergic uncontrolled asthma. Furthermore, in 2014, the FDA and EMA endorsed the drug for the treatment of spontaneous chronic urticaria in adult and adolescent patients (age ≥ 12 years) with inadequate response to H1-antihistamines [2, 5].

The clinical efficacy and safety of omalizumab have already been extensively demonstrated in many studies [6–29], including in pediatric patients. Several clinical trials have also explored the use of the Omalizumab in the treatment of other diseases in which IgE play a role (such as allergic rhinitis, food and drugs allergy, anaphylaxis, keratoconjunctivitis, urticaria, angioedema, allergic bronchopulmonary aspergillosis, atopic dermatitis, non-allergic asthma, Churg-Strauss syndrome, nasal polyposis, chronic rhinosinusitis, eosinophilic otitis media, bullous pemphigoid, contact dermatitis, mastocytosis) with promising results [30–32].

In this study, we present a case series of eight patients treated with omalizumab for severe persistent asthma and/or severe food allergy.

## Patients and methods

All patients were followed at the Pediatric Allergy, University Hospital “Gaetano Martino” in Messina, Italy. At the start of the study, previous and maintenance treatments, adverse events, comorbidities, specific and total IgE levels, history of emergency room accesses and hospitalizations were recorded (Table 1). A skin prick test (SPT) and asthma control test (ACT) were performed. At each follow-up visit, anthropometric and clinical data, of pulmonary function tests (PFTR), administered standard therapy together with any changes, and adverse events were recorded (Table 2). Overall data on SPT wheal diameter, total and specific IgE variation, treatment duration are reported in Table 3. Asthma diagnosis and severity were periodically reassessed according to GINA Guidelines [33]. Adverse drug reaction (ADR) to omalizumab were registered at each visit according to EMA guidelines [4].

**Table 1:** Patient clinical features and allergy test results before starting omalizumab

Patient	Age	Sex	Allergy disease	Comorbidity	Symptoms	SPT and PBP (mm)	Serum IgEs (KU/L)	Total Serum IgE (IU/L)	Maintenance Therapy	First Aid Access	Hospitalizations	ACT
1	13	M	Severe asthma	Allergic rhinitis, Eosinophilic esophagitis	Non-controlled asthma and exacerbations during HDM SLIT	DP = 9, DF = 7, Olea = 5 Milk = 8, Bos d 8 = 6 Bos d 4 = 10, Bos d 5 = 7 PBP = 10	DP = 100, DF = 82 Olea = 68 Milk = 2.14 --- ---	1003	Fluticasone 1000 mcg/die LABA 100 mcg/die LTRA 10 mg/die	3	3	8
2	15	M	Severe asthma	Allergic rhinitis, Chronic rhinosinusitis	Noncontrolled asthma and exacerbations during HDM SLIT	DP = 5, DF = 6 Pellitory = 5	DP = 100, DF = 100 Pellitory = 12	372	Fluticasone 1000 mcg/die LABA 100 mcg/die LTRA 10 mg/die	3	3	14
3	10	M	Moderate-severe asthma Severe Cow’s milk allergy	/	Anaphylactic reaction with traces cow’s milk	DP = 5, DF = 4 Pellitory = 5, Cat = 4 Milk = 16, Bos d 4 = 13, Bos d 5 = 11, Bos d 8 = 12	DP = 23.4, DF = 17.1 Pellitory =20.7, Cat = 9.51, Milk > 100, Bos d 8 = 100, Bos d 4 = 92, Bos d 5 = 38.6	668	Fluticasone 500 mcg/die LABA 100 mcg/die	2	2	16
4	9	F	Severe asthma	Pnx, Pnm	Non-controlled asthma and frequent exacerbations PNX	DP = 5, DF = 3	DP = 62.8, DF = 31.3	280	Fluticasone 500 mcg/die LABA 100 mcg/die LTRA 10 mg/die	5	5	10
5	7	F	Severe Cow’s milk allergy	/	Anaphylaxis after 4 ml of cow’s milk	Milk = 7 Bos d 8 = 7 Bos d 4 = 6 Bos d 5 = 5	Milk = 20.9 Bos d 8 = 18.1 Bos d 4 = 5.73 Bos d 5 = 3.49	68.8	OIT	4	4	/
6	14	M	Moderate-severe asthma Severe cow’s milk allergy	/	Asthma exacerbation after cow’s milk in traces	DP = 4, DF = 3 Cat = 4, Dog = 5 Milk = 8, PBP milk = 11 Bos d 4 = 13.5 mm, Bos d 5 = 8 mm, Bos d 8 = 11 mm	DP = 1.18, DF = 1.12 Cat = 1, Dog 3.21 Milk = 90, Bos d 8 = 62.3 Bos d 4 = 62.5 Bos d 5 = 36.6	597	Fluticasone 1000 mcg/die LABA 100 mcg/die	6	6	12
7	8	M	Severe cow’s milk allergy	/	Anaphylaxis after cow’s milk in traces	Milk 7 Bos d 8 = 12 Bos d 4 = 6 Bos d 5 = 11 PBP milk = 15	Milk 25.1 Bos d 8 = 16 Bos d 4 = 5.99 Bos d 5 = 4.24	79.9	OIT	1	1	/
8	11	M	Moderate-severe asthma Severe cow’s milk allergy	/	Anaphylaxis after cow’s milk in traces	DP = 4, DF = 5 Pellitory = 5, Cat = 5 Milk = 16, Bos d 8 = 18 Bos d 4 = 11, Bos d 5 = 12 PBP milk = 17	DP = 17.1, DF = 23.4 Pellitory = 20.7, Cat = 9.51 Milk > 100, Bos d 8 > 100 Bos d 4 = 15.8 Bos d 5 = 9.26	972	Fluticasone 500 mcg/die LABA 100 mcg/die	4	4	14

*Abbreviations: ACT* asthma control test, *DP Dermatophagoides pteronyssinus*, *DF Dermatophagoides farinae*, *HDM* house dust mite, *IgE* immunoglobulin E, *LABA* long acting beta_2_agonist, *LTRA* leukotriene receptor antagonist, *OIT* oral immunotherapy, *PBP* prick by prick, *Pnm* pneumomediastinum, *Pnx* pneumothorax, *SLIT* sublingual specific immunotherapy, *SPT* skin prick test

**Table 2:** Asthma standard therapy and FEV_1_ before omalizumab (T0) and after 1 month (T1), 3 months (T3), 6 months (T6), 12 months (T12)

Patient	Age	Omalizumab	Duration of treatment (weeks)	T0		T1		T3		T6		T12		First Aid Access	Hospitalization	ACT
				Maintenance Treatment	FEV_1_, %	Maintenance Treatment	FEV_1_, %	Maintenance Treatment	FEV_1_, %	Maintenance Treatment	FEV_1_, %	Maintenance Treatment	FEV_1_, %			
1	11	375 mg every 2 weeks	78	Fluticasone 1000 mcg/die LABA 100 mcg/die LTRA 10 mg/die	70	Fluticasone 1000 mcg/die LABA 100 mcg/die LTRA 10 mg/die	70	Fluticasone 500 mcg/die LABA 100 mcg/die	75	Fluticasone 250 mcg/die	86	Suspended	99	1	1	20
2	15	450 mg every 4 weeks	96	Fluticasone 1000 mcg/die LABA 100 mcg/die LTRA 10 mg/die	70	Fluticasone 500 mcg/die LABA 100 mcg/die LTRA 10 mg/die	70	Fluticasone 200 mcg/die LABA 100 mcg/die	73	Fluticasone 200 mcg/die	76	Suspended	101	1	1	25
3	10	300 mg every 4 weeks	16	Fluticasone 500 mcg/die LABA 100 mcg/die	73	Fluticasone 500 mcg/die LABA 100 mcg/die	73	Fluticasone 200 mcg/die LABA 100 mcg/die	75	Fluticasone 200 mcg/die	80	Suspended	101	1	1	20
4	9	150 mg every 4 weeks	104	Fluticasone 500 mcg/die LABA 100 mcg/die LTRA 10 mg/die	61	Fluticasone 500 mcg/die LABA 100 mcg/die LTRA 10 mg/die	67	Fluticasone 500 mcg/die LABA 100 mcg/die	73	Fluticasone 200 mcg/die LABA 100 mcg/die	79	Fluticasone 200 mcg/die	85	2	2	20
5	7	75 mg every 4 weeks	16	–	/	–	/	OIT	/	OIT	/	Milk desensitiz-ation	/	/	/	NO
6	14	450 mg every 4 weeks	48	Fluticasone 1000 mcg/die LABA 100 mcg/die	73	Fluticasone 1000 mcg/die LABA 100 mcg/die	73	Fluticasone 500 mcg/die LABA 100 mcg/die	75	Fluticasone 250 mcg/die	80	Suspended	100	2	2	20
7	8	75 mg every 4 weeks	16	–	/	–	/	OIT	/	OIT	/	Milk desensitiz-ation	/	/	/	NO
8	10	300 mg every 2 weeks	48	Fluticasone 500 mcg/die LABA 100 mcg/die	76	Fluticasone 500 mcg/die LABA 100 mcg/die	76	Fluticasone 250 mcg/die LABA 100 mcg/die	76	Fluticasone 250 mcg/die	80	Fluticasone 200 mcg/die	89	2	2	20

*Abbreviations: ACT* asthma control test, *FEV*_*1*_ forced expiratory volume in 1 s, *LABA* long acting β_2_ agonist, *OIT* oral immunotherapy

**Table 3:** Duration of omalizumab therapy with total IgE and sIgE before and after treatment

Patient	Duration of treatment (weeks)	Total Serum IgE (IU/L)		Serum IgEs (KU/L)	
		Before treatment	After treatment	Before treatment	After treatment
1	78	1003	490	DP = 100 DF = 82 Olive = 68 Milk = 2.14	DP = 59.3 DF = 36.3 Olive = 1.41
2	96	372	760	DP = 100 DF = 100 Pellitory = 12	DP = 100 DF = 100 Pellitory = 3.85
3	16	668	309	Milk > 100 Bos d 8 = 100 Bos d 4 = 15.8 Bos d 5 = 9.26	Milk > 100 Bos d 8 > 100 Bos d 4 = 20.3 Bos d 5 = 8.4
4	104	280	126	DP = 62.8 DF = 31.3	DP = 70 DF = 30.9
5	16	68.8	35	Milk = 20.9 Bos d 8 = 18.1 Bos d 4 = 5.73 Bos d 5 = 3.49	Milk = 8.93 Bos d 8 = 5.18 Bos d 4 = 4.09 Bos d 5 = 1.6
6	48	597	930	DP = 1.18 DF = 1.12 Milk = 90 Bos d 8 = 62.3 Bos d 4 = 62.5 Bos d 5 = 36.6	Der. P 0.25 Der. F 0.13 Milk > 100 Bos d 8 = 100 Bos d 4 > 100 Bos d 5 = 69.3
7	16	79.9	71	Milk = 25.1 Bos d 8 = 16 Bos d 4 = 5.99 Bos d 5 = 4.24	Milk = 9 Bos d 8 = 4.06 Bos d 4 = 2.93 Bos d 5 = 1.61
8	48	972	309	DP = 17.1 DF = 23.4 Pellitory = 20.7 Cat = 9.51 Milk > 100 Bos d 8 > 100 Bos d 4 = 15.8 Bos d 5 = 9.26	DP = 2.54 DF = 25.9 Pellitory = 18.1 Cat = 4.9 Milk > 100 Bos d 8 > 100 Bos d 4 = 3.87 Bos d 5 = 2.4

*Abbreviation: IgE* immunoglobulin E

All parents signed a written informed consent on behalf of their child. Patients with food allergy were treated with omalizumab off-label. Therefore, the study design was submitted to and approved by the Ethics Committee of University.

## Case presentations

### Case 1

A 13-year-old boy had suffered from severe allergic asthma since preschool age. He had house dust mite (HDM) and olive pollen allergy detected by SPT (Dermatophagoides pteronyssinus [DP] 9 mm, Dermatophagoides farinae [DF] 7 mm and Olive pollen 5 mm) and specific IgE levels (100, 82 and 68 IU/mL, respectively), with high total IgE (1003 IU/mL) levels and normal eosinophil count (110/mmc) (Table 1). The pulmonary function tests (PFTs) were constantly abnormal, with a forced expiratory volume in 1 s (FEV1) < 80% of the predicted value before bronchodilation. He had poor disease control, despite daily high-dose ICS plus inhaled LABA and leukotriene receptor antagonist (LTRA), previous sublingual specific immunotherapy (SLIT) with dust mite extract (continuously for 3 years from the age of 5), during which he experienced many exacerbations (Table 2).

He also suffered from the age of 8 years from mild persistent allergic rhinitis treated with intranasal corticosteroids and eosinophilic esophagitis (EoE) confirmed by endoscopic and histological findings.

In this context, SPT was performed and revealed a sensitization to milk and soy (milk extract 8 mm; Bos d 4 = 10 mm; Bos d 5 = 7 mm; Bos d 8 = 6 mm; Prick by prick [PBP] with fresh milk 10 mm and soy [5 mm]). The first approach to EoE included a semi-elemental diet and a strict soy/milk avoidance which lead to symptomatic and histological relief. Due to the poor palatability, he suspended the diet after three months.

When he was 11 years old, he experienced worsening asthmatic symptoms, requiring emergency room access and hospitalizations. Therefore, he started therapy with omalizumab (375 mg subcutaneously) every 2 weeks, according to the reference nomogram for body weight and total IgE level [4]. Overall the patient received omalizumab for 18 months with clinical and spirometric improvement. After three months of therapy, the long-term treatment with high-dose ICS plus inhaled LABA and LTRA was gradually reduced until its suspension, at the 12th month of therapy with omalizumab (Table 2). According to Global Initiative for Asthma (GINA)-guided assessment [33], he continued to have good asthma control over time with normal spirometric parameters (FEV1 99%, forced expiratory flow at 25–75% of the pulmonary volume [FEF25–75] 70%). Furthermore, he showed a reduction of the average wheal diameter for mites and olive tree (DP 6 mm, DF 5 mm, olive 3 mm) serum IgE (sIgE) (DP 59.3 KU/L, DF 36.3 KU/L, olive tree 1.41 KU/Ll) with lower total IgE (490 IU /mL) (Table 3).

The treatment response in EoE was assessed by evaluating the change in clinical symptoms score (Pediatric Eosinophilic Esophagitis Symptom Score [PEESS® v2.0]) and by endoscopy with biopsy findings [34, 35]. There was a non-significant histological change during omalizumab therapy despite a mild decrease in symptom score. Therefore, he started oral viscous budesonide (BUD) with clinical and histological relief but recurrence of symptoms when steroids were discontinued.

### Case 2

A 15-year-old boy presented with severe allergic asthma, moderate persistent allergic rhinitis, and chronic rhinosinusitis. The rhinosinusitis symptoms were quite troublesome (visual analog scale [VAS] = 5) despite long-term therapy with nasal saline irrigation, oral antibiotic ≥12 weeks and intranasal corticosteroids. During the work-up and according to the EPOS guidelines, cystic fibrosis, primary ciliary dyskinesia, immunodeficiencies, anatomical abnormalities and nasal polyposis were excluded [36].

Asthma appeared in preschool age but worsened in the recent years; it was not controlled by daily high-dose ICS, inhaled LABA and LTRA. He had HDM and pellitory allergy detected by SPT (DP 5 mm, DF 6 mm and pellitory 5 mm) with sIgE levels (100, 100 and 12 IU/mL, respectively) and high total IgE (372 IU/mL). Spirometry showed a persistent reversible airflow obstruction pattern (FEV1 70%, FEF25–75 < 50%). A SLIT with HDM was attempted when he was 7 years old but discontinued because of asthma exacerbations (Table 1).

Therefore, he started omalizumab (450 mg subcutaneously) every 4 weeks, according to the reference nomogram [4]. After nine months of therapy, he achieved better asthma control with FEV1 > 80% and started the ICS + LABA treatment step-down until its suspension. He underwent omalizumab therapy for a total of 24 months, at the end of which he showed asthma remission and normal spirometric values (FEV1 98%, FEF25–75 73%). At the end of the treatment, total IgE were 760 IU/mL and sIgE DP 100 KU/L, DF 100 KU/L, pellitory 3.85 KU/L (Tables 2 and 3). Under omalizumab he also experienced an improvement in symptoms (VAS = 2) and clinical signs of chronic rhinosinusitis, suggesting the role of IgE in the pathogenesis of the mucosal inflammation as suggested by other studies [37].

### Case 3

A 10-year-old boy with severe cow’s milk allergy (CMA) and moderate-severe allergic asthma was followed at our allergy outpatient clinic.

Asthmatic symptoms, which started at the age of 4 years, were controlled at first by ICS at medium dosage and LTRA, and then from high-dose ICS (fluticasone up to 500 mcg/day) + LABA, with an FEV1 of 76%. He had HDM, pellitory and cat allergy confirmed by SPT (DP 5 mm, DF 4 mm, pellitory 5 mm, cat dander 4 mm) with sIgE levels (DP 23.4, DF 17.1, pellitory 20.7, cat dander 9.51 KU/L) and high total IgE (668 IU/mL) (Table 1).

The diagnosis of CMA was made at the age of 2 months and confirmed at further stages through SPT (milk 16 mm, Bos d 4 = 13 mm, Bos d 5 = 11 mm, Bos d 8 = 12 mm), sIgE (milk > 100 KU/L, Bos d 8 > 100 KU/L, Bos d 4 = 15.8 KU/L, Bos d 5 = 9.26 KU/L) and oral food challenge (OFC) (Table 3).

Spontaneous remission of CMA did not occur over time, and the child experienced four anaphylactic adverse reactions from accidental ingestion of foods containing hidden milk proteins and bronchospasm after inhalation of powder containing cow’s milk proteins. At the age of 10, after achieving better asthma control, he started oral immunotherapy (OIT) to milk after a protocol already tested at our Centre [38] failed because of anaphylaxis after 0.5 mL of milk.

Therefore, on the basis of recent evidence, he received omalizumab in combination with oral milk desensitization [39]. The protocol consisted of three steps:

1. Nine weeks of pre-treatment with omalizumab (300 mg subcutaneously every two weeks according to the reference nomogram).
2. A second phase (from 9th to 16th week) characterized by the association of omalizumab and OIT. Oral cow’s milk desensitization was performed in 2 phases: a rush phase on the first day (starting with 5 drops of milk, with increasing doses every 30 min to a maximum of 30 mL corresponding to 1000 mg of cow’s milk proteins) and a slow phase with weekly increases in the dose of milk which, if tolerated, was taken at home.
3. A “consolidation” phase (from 17th to 24th week) in which omalizumab was discontinued and the patient carried out only OIT with a weekly visit to the Day-Hospital.

He presented an excellent response, achieving 140 mL of milk during the third phase.

Unfortunately, two weeks after a viral, milk consumption in the same amounts previously tolerated triggered life-threatening anaphylaxis requiring hospitalization. This adverse reaction was probably worsened by infection, as reported in the literature [40]. The patient’s parents decided to discontinue OIT and resume a cow’s milk protein-free diet. Evaluations performed after 6 months showed total IgE = 309 IU/mL with milk sIgE > 100 KU/L, Bos d 8 > 100 KU/L, Bos d 4 = 20.3 KU/L, Bos d 5 = 8.4 KU/L (Table 3).

Regarding allergic asthma, omalizumab therapy allowed the step-down of ICS + LABA (starting from the 12th week of anti-IgE treatment) until achieving its suspension. He presented normal lung function parameters with sIgE DP and DF > 100 KU/L (Table 1).

### Case 4

A 9-year-old girl was admitted to the intensive care unit with acute respiratory failure (ARF), pneumothorax (PNX) and pneumomediastinum after a serious asthma attack. Asthma severity was underestimated and the symptoms undertreated, despite being diagnosed with allergic asthma at the age of 5 (SPT DP 5 mm, DF 3 mm, sIgE DP 62.8, DF 31.3 KU/L and total IgE 280 IU/mL) (Table 1). At admission, chest radiography and computed tomography (CT) scan documented bilateral apical PNX, pneumomediastinum, and subcutaneous emphysema extended to the soft tissues of the thorax and neck.

After resolution of the ARF, the main causes of spontaneous secondary PNX and pneumomediastinum (such as congenital malformations, foreign body inhalation and/or toxic substances, cystic fibrosis, trauma, pneumonia, interstitiopathies) were reviewed, confirming the relationship with severe uncontrolled chronic asthma (FEV1 61% of predicted).

The patient started high-dose ICS (fluticasone 500 mcg/die) plus LABA (100 mcg/die) and LTRA (10 mg/die), achieving partial control as shown by ACT and spirometry at 4, 8 and 12 weeks (Table 2). Therefore, she started omalizumab (150 mg subcutaneously every 4 weeks), according to the reference nomogram [4] for 24 months. During follow-up, there was a gradual improvement in respiratory performances and inflammation conditions, shown by spirometry (3rd month: FEV1 67%, 6th month: FEV1 73%, 6th month: FEV1 79%, 12th month: FEV1 85%) and reduction in exhaled nitric oxide (eNO) before and after treatment (35 vs 8 ppb). A reduction of total IgE (126 IU/mL) was also observed with sIgE almost unchanged (DP 70, DF 30.9 KU/mL) (Table 3). At the same time, it was possible to proceed with the gradual step down of inhalation therapy (from high-dose ICS + LABA + LTRA to low-dose ICS) (Table 2).

### Case 5

A 7-year-old girl was referred to the allergy outpatient Clinic for severe CMA, dating from the first year of life. Food allergy was confirmed by SPT (milk 7 mm, Bos d 4 = 6 mm, Bos d 5 = 5 mm, Bos d 8 = 7 mm), sIgE (milk 20.9 KU/L, Bos d 8 = 18.1 KU/L, Bos d 4 = 5.73 KU/L, Bos d 5 = 3.49 KU/L) and a positive OFC (resulting in anaphylaxis after 4 mL of fresh milk) (Table 1). At the age of 6, she started OIT to milk following a protocol already tested at our Centre [38], achieving the dose of 35 mL after 12 months of therapy. However, she presented with anaphylaxis after 40 mL of milk, resulting in interruption of OIT. A year later, following the pioneering work of Nadeau et al. [39] she started desensitization to milk in combination with omalizumab therapy (75 mg every 4 weeks according to the reference nomogram [4]) following the protocol already described for patient number 3. The girl was able to tolerate ordinary daily milk amounts, but after 4 weeks of food avoidance due to personal considerations, she presented an adverse reaction characterized by urticaria and angioedema after the introduction of 30 mL of milk. She restarted rush OIT lasting 2 days, after which she reintroduced milk into her diet without any adverse reaction. Two months after stopping OIT and 4 months from the last omalizumab injection, she is introducing milk and dairy products into her diet at least several days per week.

Alongside milk desensitization, there was a decline in total IgE, sIgE (milk 8.93 KU/L, Bos d 8 = 5.18 KU/L, Bos d 4 = 4.09 KU/L, Bos d 5 = 1.6 KU/L, total IgE 35 IU /mL) and SPT mean wheal diameter (milk 4 mm, Bos d 8 = 4 mm, Bos d 4 = 3 mm, Bos d 5 = 2 mm) (Table 3).

### Case 6

A 14-year-old boy with severe CMA and moderate-severe allergic asthma was followed at our allergy outpatient clinic.

His respiratory symptoms started in preschool, then he developed allergy to HDM, cat and dog (SPT mean wheal diameter DP 4 mm, DF 3 mm, cat 4 mm, dog 5 mm, sIgE DP 1.18 KU/L, DF 1.12 KU/L, cat 1 KU/L, dog 5.36 KU/L, with total IgE 597 IU/mL). Asthma was controlled by high dose ICS plus LABA (Table 1).

CMA was diagnosed at the age of 10 months, confirmed by SPT (milk 8 mm, Bos d 4 = 13.5 mm, Bos d 5 = 8 mm, Bos d 8 = 11 mm, PBP 11 mm), sIgE (milk 90 KU/L, Bos d 8 62.3 KU/L, Bos d 4 62.5 KU/L, Bos d 5 = 36.6 KU/L), and OFC performed at pre-established intervals (Table 1). He followed a milk and dairy free diet but presented two anaphylaxis episodes from accidental exposure to milk at the age of six and eight years.

OIT was attempted at the age of 13 but was unsuccessful due to adverse reactions and poor compliance. A year later he underwent OIT plus omalizumab (450 mg every 4 weeks according to the reference nomogram) [4].

The experimental protocol was modified a little from the one given above, and characterized by:

1. First step: (0–8 weeks) pre-treatment with omalizumab.
2. Second step: (8–48 weeks) combined therapy (omalizumab plus OIT) with a rush desensitization phase lasting 2 days and a slow phase with a monthly dose increase (25% at a time) in the outpatient clinic and continuing daily home intake of the maximum tolerated milk amount.
3. Third step: (48–60 weeks) only OIT and subsequently free diet with monthly follow-up.

Although anti-IgE therapy was prolonged for a total of 48 weeks, milk desensitization failed. From the second month of combined therapy, the patient experienced a mild-to-moderate adverse reaction slowing escalation of the milk dose. At the 52nd week of therapy, he presented with anaphylaxis after 10 mL of milk and the study protocol was interrupted. This lack of success was also probably due to the poor family adherence; in fact, the patient did not regularly take milk at home, and sometimes the outpatient appointments were postponed. A year after stopping the study protocol, an increase both in total IgE (930 IU/mL) and sIgE (milk> 100 KU/L, Bos d 8 > 100 KU/L, Bos d 4 > 100 KU/L, Bos d 5 = 69.3 KU/L) was observed. However, during therapy with omalizumab, he showed clinical improvement of asthma symptoms and respiratory function parameters (FEV1 80%) with the chance to start the step-down of inhalation therapy (from the third month of treatment) (Table 2). Currently, the boy has mild allergic asthma well controlled by low-dose ICS and continues a milk- and dairy-free diet.

### Case 7

Severe CMA affected an eight-year-old boy from the age of nine months. A last positive OFC was performed at the age of two years, and then he continued a cow’s milk proteins-free diet. He suffered from anaphylaxis at the age of 5 because of accidental exposure to milk. During his first outpatient visit, CMA was confirmed by SPT (milk 7 mm, Bos d 4 = 12 mm, Bos d 5 = 6 mm, Bos d 8 = 11 mm, PBP 15 mm), sIgE (milk 25.1 KU/L, Bos d 8 = 16 KU/L, Bos d 4 = 5.99 KU/L, Bos d 5 = 4.24 KU/L, with total IgE 79.9 IU/mL), and a positive OFC (anaphylaxis after 3 mL of milk) (Table 1).

Milk OIT was proposed and started without success because anaphylaxis occurred at 1.5 mL of milk.

Therefore, on the basis of scientific evidence [39] in a patient at high risk of serious food adverse reactions and refractory to traditional OIT [41], omalizumab-assisted desensitization to milk was proposed and started.

The oral desensitization protocol to milk was similar to the one already described for subject number 3 and included pre-treatment with omalizumab (75 mg subcutaneously every 4 weeks for 9 weeks according to reference nomogram [4]), a second phase of combined therapy (omalizumab plus OIT) lasting 7 weeks and finally a third step during which the subject only underwent milk OIT. The results of the study protocol were exceptional, and the boy was able to include milk and dairy products into the diet at least several days per week without any adverse event.

Moreover, a reduction of total and specific IgE levels was observed (total IgE 71 IU/mL, milk 9 KU/L, Bos d 8 = 4.06 KU/L, Bos d 4 = 2.93 KU/L, Bos d 5 = 1.61 KU/L) (Table 3).

### Case 8

An 11-year-old boy with allergic moderate-severe asthma, mild-moderate persistent rhinitis, and severe CMA was followed at our allergy outpatient clinic.

He had HDM, pellitory pollen and cat dander allergy confirmed by SPT (DP 4 mm, DF 5 mm, *Parietaria judaica* 5 mm, cat dander 5 mm) and sIgE (17,1 KU/L, 23.4 KU/L, 20.7 KU/L, and 9.51 KU/L respectively), with high total IgE (972 IU/mL) levels. Asthma was not well controlled by ICS (fluticasone 500 mcg/die) plus LABA, while rhinitis was treated with nasal corticosteroids and oral antihistamines.

CMA was diagnosed at the age of 3 months and persisted over the years. The patient experienced 4 episodes of anaphylaxis, after accidental exposure to traces of cow’s milk proteins, needing hospitalization. When he was 8-years old, he started conventional OIT at another Allergy Clinic, following a different protocol [42]. During the rush phase, he presented with anaphylaxis after 3 mL of milk diluted in 20 mL of water and OIT was discontinued.

At the age of 10, after reviewing SPT (milk 16 mm, Bos d 8 = 6 mm, Bos d 4 = 16 mm, Bos d 5 = 21 mm) we tried to restart conventional OIT [38] with bad results (anaphylaxis after 4 drops of milk).

After a year, we reassessed allergy tests (SPT: Milk = 16 mm, Bos d 4 = 11 mm, Bos d 5 = 12 mm, Bos d 8 = 18 mm, PBP 17 mm; sIgE: milk > 100 KU/L, Bos d 8 > 100 KU/L, Bos d 4 = 15.8 KU/L, Bos d 5 = 9.26 KU/L) and started omalizumab-assisted desensitization to milk.

He received omalizumab (300 mg every 4 weeks according to the reference nomogram [4]) and OIT following the same protocol as patient number 6. He was able to drink 180 mL of milk after stopping omalizumab but experienced anaphylaxis again during a viral pharyngitis, and the protocol was interrupted.

Asthma exacerbations reduced and spirometry improved (pre-FEV1 76% vs. post 89%) during anti-IgE therapy. Therefore he started step-down of inhalation therapy (currently he is on low-dose ICS).

### Safety

In total, all of subjects in our series presented at least one ADR during therapy. The most commonly reported ADRs (*N* = 7), were local reactions (pain, erythema, and edema at the site of injection). Other adverse events were also reported, such as asthenia (*N* = 1), fever (*N* = 1), headache (*N* = 4), and generalized malaise (*N* = 1) after drug administration. None of these adverse events required discontinuation of treatment or hospitalization. No case of IgE-mediated adverse reaction or anaphylaxis has been reported. Regarding OIT, many adverse food reactions were registered, as reported in Table 4.

**Table 4:** Safety profile of omalizumab-assisted oral immunotherapy

Patient	Symptoms	Concomitant Trigger	Milk (mL)	OIT step	Emergency treatment	Outcome
3	Rhinitis	No	120	18th week (OIT)	Antihistamine	Failed
	Rhinitis and asthma	Acute illness	100	20th week (OIT)	Antihistamine and Salbutamol	
	Anaphylaxis	Acute illness	140	22th week (OIT)	Adrenaline OIT discontinued	
5	No	NA	NA	NA	NA	Desensitization
6	Urticaria and sickness	No	18	12th week (O + OIT)	Antihistamine Corticosteroids	Failed
	Urticaria	No	7	15th week (O + OIT)	Antihistamine	
	Urticaria	No	4	20th week (O + OIT)	Antihistamine	
	Anaphylaxis	No	10	52th week (OIT)	Adrenaline OIT discontinued	
7	No	NA	NA	NA	NA	Desensitization
8	Anaphylaxis	Acute illness	150	52th week (OIT)	Adrenaline OIT discontinued	Failed

*Abbreviations: NA* not applicable, *OIT* oral immunotherapy

## Discussion

Of our series of eight subjects, six had moderate-severe allergic asthma and, among these, four were not controlled or were only partially controlled by high-dose ICS + LABA and had a significant reduction in FEV1. Five patients had other comorbidities (IgE-mediated food allergy, EoE, allergic rhinitis and rhinosinusitis). Two subjects had only severe food allergy.

In the asthmatic patients, therapy with omalizumab ranged from 16 to 104 weeks, but two subgroups could be identified: those treated only for allergic asthma (Group A, *N* = 3) and those who also had a food allergy (Group B, *N* = 3). Omalizumab therapy in the three patients with moderate-severe asthma and concomitant IgE-mediated CMA was started with the dual purpose of achieving better asthma control and promoting milk desensitization.

Patients in Group A had severe allergic asthma not controlled by standard therapy (GINA STEP 4/5) [33]. All subjects achieved good asthma control after starting anti-IgE therapy (Table 2). We proceeded to gradually step down standard therapy, with an ICS minimum reduction of 50% until its complete suspension in two subjects. FEV1 improved at the end of the treatment in all patients (Table 2).

Subjects in Group B had moderate-severe allergic asthma partially or not controlled by standard therapy (GINA STEP 4/5) and food allergy. All subjects achieved good asthma control that allowed step down of standard therapy and PFTR improvement.

In two patients in Group B, complete suspension of standard asthma therapy was possible, despite the omalizumab therapy in this group, administered according to the oral desensitization protocol, lasting less (16–48 weeks) than in Group A (78–104 weeks) (Tables 2 and 3). Subjects also suffering from allergic rhinitis experienced an improvement in nasal symptoms during therapy with omalizumab, when evaluated by a total score of nasal symptoms (TNSS) (weekly pre-treatment range 42–10 vs. post-treatment 29–4). The subject with chronic rhinosinusitis presented a symptomatic improvement (pre-treatment VAS = 5 vs post-treatment VAS = 2).

Five patients suffered from food allergy (3 belonging to the Group B with asthma comorbidity) and 2 subjects (Group C) received the anti-IgE assisted OIT for food allergy.

According to European Guidelines, all patients underwent allergy testing and OFC before starting the desensitization protocol [43].

Omalizumab-assisted OIT had a variable duration depending on the protocol used and the emergence of adverse reactions. In Group B, two subjects achieved a maintenance milk dose of 140 mL and 180 mL, respectively, showing desensitization continued after omalizumab discontinuation. However, both subjects presented an anaphylaxis episode after taking milk a few days after a viral infection, suggesting the role of infectious diseases as a trigger disrupting desensitization. The third subject suspended the omalizumab-assisted OIT after about 12 months because of anaphylaxis after the administration of 10 mL of milk. However, in this case, adherence to therapy was not adequate, as regularity of home milk intakes had not been followed.

In Group C, subjects were successfully desensitized to milk, but we cannot predict if they will be tolerant lifelong. Furthermore, a reduction of total and specific IgE (pre vs. post-treatment) was observed in the two subjects who successfully completed the desensitization protocol.

This report suggests that omalizumab is an excellent therapeutic option in subjects with severe allergic asthma that remains uncontrolled despite high-dose ICS plus other controller medications, as already widely verified in other studies.

These findings suggest the potential role of omalizumab to support oral desensitization in patients with IgE mediated food allergy. It could promote a reduction in adverse immune response to food, along with a lower risk of anaphylaxis and increased quality of life for children and their families. However, further studies are needed to confirm these promising results. Currently, omalizumab is not approved for the treatment of food allergy. No efficacy was seen in EoE which has more complex pathogenesis. In addition, we observed a clinical efficacy in treating allergic rhinitis and rhinosinusitis. TNSS scores valuating nasal symptoms (blockage, rhinorrhea, itching and sneezing) in patients with allergic rhinitis were reduced thus suggesting the role of omalizumab also on rhinitis-related symptoms. In conclusion omalizumab represents the pivotal treatament option for severe, uncontrolled asthma in childhood. It could promote an effective treatment of IgE mediated food allergy,and severe allergic rhinitis. However, large and well controlled studies are awaited to confirm these promising results.

## Abbreviations

ACT: Asthma control test
ADR: Adverse drug reaction
ARF: Acute respiratory failure
BUD: Budesonide
CMA: Cow’s milk allergy
CT: Computed tomography
DF: Dermatophagoides farinae
DP: Dermatophagoides pteronyssinus
EMA: European Medicines Agency
EoE: Eosinophilic esophagitis
FDA: Food and Drug Administration
FEV1: Forced expiratory volume in 1 s
HDM: House dust mite
ICS: Inhaled corticosteroids
Ig: Immunoglobulin
LABA: Long-acting β2-agonist
LTRA: Leukotriene receptor antagonist
mAb: Monoclonal antibody
OFC: Oral food challenge
OIT: Oral immunotherapy
PBP: Prick by prick
PEESS: Pediatric Eosinophilic Esophagitis Symptom Score
PFTR: Pulmonary function tests
PNX: Pneumothorax
sIgE: Serum IgE
SLIT: Sublingual specific immunotherapy
SPT: Skin prick test
TNSS: Total score of nasal symptoms
VAS: Visual analog scale
